# Prognostic and predictive value of PDL1 expression in breast cancer

**DOI:** 10.18632/oncotarget.3216

**Published:** 2014-12-31

**Authors:** Renaud Sabatier, Pascal Finetti, Emilie Mamessier, José Adelaide, Max Chaffanet, Hamid Raza Ali, Patrice Viens, Carlos Caldas, Daniel Birnbaum, François Bertucci

**Affiliations:** ^1^ Département d'Oncologie Moléculaire, “Equipe labellisée Ligue Contre le Cancer”, Centre de Recherche en Cancérologie de Marseille (CRCM), Institut Paoli-Calmettes, INSERM UMR1068, CNRS UMR725, Marseille, France; ^2^ Département d'Oncologie Médicale, CRCM, Institut Paoli-Calmettes, Marseille, France; ^3^ Faculté de Médecine, Aix-Marseille Université, Marseille, France; ^4^ Department of Pathology, University of Cambridge, Cambridge, United Kingdom; ^5^ Cancer Research UK Cambridge Institute, University of Cambridge, Cambridge, United Kingdom

**Keywords:** breast cancer, chemotherapy, immune response, PDL1, survival

## Abstract

Expression of programmed cell death receptor ligand 1 (PDL1) has been scarcely studied in breast cancer. Recently PD1/PDL1-inhibitors have shown promising results in different carcinomas with correlation between PDL1 tumor expression and responses. We retrospectively analyzed *PDL1* mRNA expression in 45 breast cancer cell lines and 5,454 breast cancers profiled using DNA microarrays. Compared to normal breast samples, *PDL1* expression was upregulated in 20% of clinical samples and 38% of basal tumors. High expression was associated with poor-prognosis features (large tumor size, high grade, ER-negative, PR-negative, *ERBB2-*positive status, high proliferation, basal and *ERBB2*-enriched subtypes). *PDL1* upregulation was associated with biological signs of strong cytotoxic local immune response. *PDL1* upregulation was not associated with survival in the whole population, but was associated with better metastasis-free and overall specific survivals in basal tumors, independently of clinicopathological features. Pathological complete response after neoadjuvant chemotherapy was higher in case of *PDL1* upregulation (50% *versus* 21%). In conclusion, *PDL1* upregulation, more frequent in basal breast cancers, was associated with increased T-cell cytotoxic immune response. In this aggressive subtype, upregulation was associated with better survival and response to chemotherapy. Reactivation of dormant tumor-infiltrating lymphocytes by PDL1-inhibitors could represent promising strategy in PDL1-upregulated basal breast cancer.

## INTRODUCTION

Despite recent progresses, nearly 20% of patients with breast cancer still develop metastases and die from disease progression. During the last decades, molecular alterations involved in mammary oncogenesis and metastatic progression have been identified, leading to major therapeutic progresses such as hormone therapy targeting the estrogen receptor (ER) and targeted therapies directed against oncogenic proteins (ERBB2, EGFR, VEGF, and PI3K/AKT/mTOR pathway). Nevertheless, if not present initially, resistant clones emerge in most of cases because of the high mutagenic and adaptable capacity of cancer cells, making the tumor responses temporary. Thanks to the adaptability of the immune response, cancer immunotherapy can theoretically address this issue. Breast cancer is less immunogenic than melanoma or renal cell carcinoma and the results of adoptive immunotherapy (interleukin 2, interferons, and vaccines) have been relatively disappointing. However, the role of immunity has emerged during the last decade with the demonstration of a favorable prognostic impact of the presence of tumor-infiltrating lymphocytes (TILs) [1-3] and of gene expression signatures of immune response, notably for ER-negative, highly proliferative tumors [4-8].

Immune response represents a complex phenomenon based on a balance between activator and inhibitor pathways that regulate TILs activity. This balance may be disturbed in certain pathological conditions such as cancer where the inhibition of the immune system will favor tumor progression. One key inhibitor is the PD1-PDL1 pathway. PD1 (Programmed cell Death 1) is a cell surface membrane protein expressed by various immune cells including T-cells; it is activated by its ligands PDL1 and PDL2, which are expressed by antigen-presenting cells such as macrophages or B-cells. After engagement by its ligands, PD1 attenuates lymphocyte activation [9-13] and promotes T-regulatory cell development and function, allowing to terminate the immune response. Recent works have suggested that it could be a main actor in cancer progression through anti-cancer immune response inhibition [10, 14, 15]. Indeed, tumor cells from different locations express PDL1 and thus can inhibit the immune response. Clinical trials testing anti-PD1 or anti-PDL1 drugs to restore anti-cancer immunity have shown very promising results with durable responses, notably in melanoma and renal, lung, prostate and bladder carcinomas [16-18], and phase III studies are ongoing. Furthermore, relationship between PDL1 expression on tumor and/or immune cells and objective response has been reported [16, 17, 19-21].

PDL1 expression has been studied in different cancers such as kidney, lung, pancreas, esophagus, ovary, colorectal, head and neck and squamous cell carcinomas, melanomas and gliomas [22-32], with evidence of correlations with clinicopathological tumor features in several studies. In breast cancer, the PD1-PDL1 pathway has been very scarcely studied [33-39]; only two prognostic studies, including 650 cases analyzed at the protein level (immunohistochemistry, IHC) [39] and 398 cases at the mRNA level [36], recently addressed the prognostic issue, but provided divergent results.

Here, we have analyzed *PDL1* mRNA expression in 45 breast cancer cell lines and 5,454 breast cancers profiled using DNA microarrays. We searched for correlations between *PDL1* expression and genomic and clinicopathological data, including survival and response to chemotherapy.

## RESULTS

### PDL1 expression and copy number alterations in breast cancer

**Figure 1:**
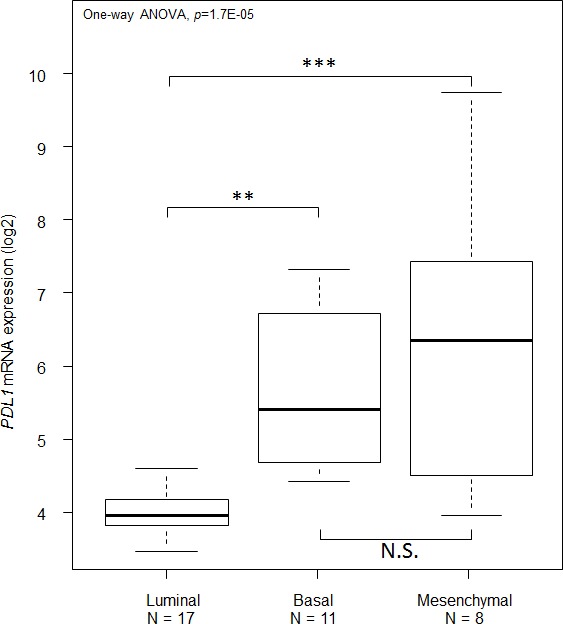
PD-L1 mRNA expression across molecular subtypes of breast cancer cell lines *PDL1* expression level reported as a box plot according to the molecular subtype of cell lines. The p-values are indicated (Tukey test) are indicated as follows: **, p<0.01; ***, p<0.001; NS, p>0.05.

*PDL1* expression was measured by using probe sets whose identity and specificity showed 100% accuracy (Supplementary Table 1). We evaluated *PDL1* expression in 45 breast cancer cell lines. Luminal cell lines (N=16) showed lower *PDL1* expression level than basal cell lines (N=11; *p*=1.46E-03, Tukey test) and mesenchymal cell lines (N=8; *p*=2.0E-04, Tukey test). *PDL1* expression was similar between basal and mesenchymal cell lines (*p*=0.34, Turkey test; Figure 1 and Supplementary Figure 1). *PDL1* expression was analyzed in 5,454 clinical breast cancer samples pooled from 18 data sets (Supplementary Tables 2-3): 1076 tumors (20%) showed *PDL1* upregulation when compared to normal breast (ratio T/NB ≥2; “PDL1-up” group), and 4378 (80%) did not show upregulation (ratio <2; “PDL1-no up” group).

Array-CGH data were available for 3,140 tumors. *PDL1* copy number alterations were rare: 134 tumors (4%) presented losses, including 13 with homozygous deletion (0.4%), whereas 163 (5%) showed gains, including 39 (1%) with amplification. Of note, 74% of amplified tumors were basal subtype. Basal tumors presented more gains when compared to the other molecular subtypes (17% *vs* from 1 to 4% for the other subtypes; *p*<1E-04, Fisher's exact test), but similar levels of losses (*p*=0.53). Correlation existed between DNA copy number and mRNA expression since tumors with *PDL1* gains displayed a higher *PDL1* expression level (*p*=2.18E-07, Student t-test; Supplementary Figure 2).

### PDL1 expression and clinicopathological features

We searched for correlations between *PDL1* mRNA expression and clinicopathological features. As shown in Table 1, *PDL1* expression was generally associated with poor-prognosis features: pathological type (with more ductal and medullary carcinoma in the “PDL1-up” group), large pathological tumor size, high tumor grade, negative ER status, negative PR status, positive ERBB2 status, and positive Ki67 status. No correlation was found with patients' age and pathological axillary lymph node status. Regarding the molecular subtypes, we observed more basal and ERBB2-enriched cases and less luminal and normal-like cases in the “PDL1-up” group than in the “PDL1-no up” group (*p*=5.0E-04, Student t-test).

**Table 1:** PDL1 expression and clinicopathological features

	PDL1- no up (N=4378), N (%)	PDL1- up (N=1076), N (%)	*p*-value
Age (years)			0.119
≤50	1021 (28%)	267 (31%)	
>50	2609 (72%)	598 (69%)	
Pathological type			5.00E-04
DUC	2315 (81%)	554 (83%)	
LOB	241 (8%)	33 (5%)	
MED	19 (1%)	37 (6%)	
MIX	109 (4%)	11 (2%)	
Other	191 (7%)	30 (5%)	
Pathological axillary lymph node status, pN			0.16
Negative	1632 (51%)	371 (48%)	
Positive	1559 (49%)	398 (52%)	
Pathological tumor size, pT			2.95E-03
pT1	1096 (42%)	211 (35%)	
pT2-T4	1517 (58%)	388 (65%)	
SBR grade			1.37E-32
1	431 (12%)	44 (6%)	
2	1544 (45%)	224 (28%)	
3	1487 (43%)	531 (66%)	
ER status			2.80E-69
Negative	1088 (25%)	564 (52%)	
Positive	3290 (75%)	512 (48%)	
PR status			3.52E-28
Negative	1925 (44%)	676 (63%)	
Positive	2406 (56%)	392 (37%)	
ERBB2 status			9.50E-04
Negative	3853 (88%)	906 (84%)	
Positive	525 (12%)	170 (16%)	
Ki67 status			5.86E-35
Negative	2586 (59%)	411 (38%)	
Positive	1789 (41%)	665 (62%)	
Molecular subtypes			5.00E-04
Luminal A	1382 (32%)	133 (12%)	
Luminal B	1057 (24%)	186 (17%)	
Basal	752 (17%)	453 (42%)	
ERBB2-enriched	614 (14%)	227 (21%)	
Normal-like	573 (13%)	77 (7%)	
Metastatic relapse			0.32
No	470 (59%)	172 (62%)	
Yes	333 (41%)	105 (38%)	
5-year MFS (%[95CI])	61% [0.58-0.65]	61% [0.55-0.67]	0.58
Death of breast cancer			1.42E-03
No	2301 (74%)	547 (80%)	
Yes	794 (26%)	136 (20%)	
5-year OSS (%[95CI])	82% [0.80-0.83]	84% [0.81-0.87]	0.07
Pathological complete response, pCR			5.80E-06
No	153 (79%)	36 (50%)	
Yes	40 (21%)	36 (50%)	

N, number of cases available; DUC, ductal carcinoma, LOB, lobular carcinoma, MIX, mixed; MED, medullary carcinoma; pN, pathological lymph node involvement; pT, pathological tumor size; MFS, metastasis-free survival; OSS, overall specific survival; pCR, pathological complete response

### PDL1 expression and immune features

Next, we investigated whether *PDL1* expression was associated with immunity-related parameters in clinical samples of the whole data set (Supplementary Table 4). First, we found a correlation between *PDL1* expression and several immune prognostic gene expression signatures of basal breast cancer [4-7]. Breast cancer samples predicted by these classifiers as having a higher expression of immune response genes (good-prognosis) indeed overexpressed *PDL1*. Second, we found that the probability of activation [40] of immune-related pathways such as IFNα, IFNγ, STAT3 and TNFα was associated with *PDL1* overexpression, both in the whole cohort of samples and in each molecular subtype (data not shown).

### PDL1 expression and metastasis-free survival

**Figure 2:**
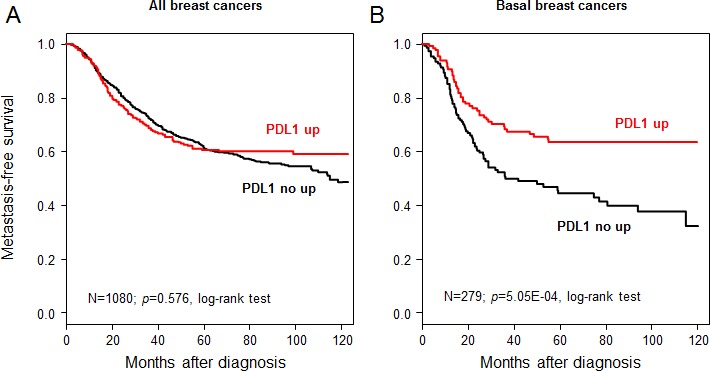
Metastasis-free survival according to PDL1 mRNA expression in the whole population and in basal breast cancers A/ Kaplan-Meier MFS curves in patients with high and low expression in the whole population. The 5-year MFS was 61% in both groups. B/ Similar to (A), but limited to patients with basal breast cancer. The respective 5-year MFS were 63 and 44%.

We assessed the prognostic value of *PDL1* expression in terms of MFS and OSS. MFS data were available for 1,080 patients, including 642 who remained metastasis-free during a median follow-up of 85 months (median MFS not reached) and 438 who displayed metastatic relapse. The 5-year MFS rate was 61% [95CI, 0.58-0.64]. In univariate analysis applied to the whole population (Table 2), axillary lymph node involvement, large tumor size, high grade, negative ER status, and negative PR status were associated with poor MFS, whereas *PDL1* expression was not (*p*=0.57, Wald test; HR=0.94 [0.75-1.17], and *p*=0.576, log-rank test, Figure 2A). The same analysis was done in each molecular subtype separately. As shown in Figure 2B, *PDL1* expression influenced MFS in the basal subtype with 63% 5-year MFS (CI95 55-73) in the “PDL1-up” group and 44% (CI95 36-54) in the “PDL1-no up” group (*p*=5.05E-04; log-rank test). By contrast, no significant influence was seen in the other subtypes (data not shown): luminal A (*p*=0.76), luminal B (*p*=0.60), ERBB2-enriched (*p*=0.09), and normal-like (*p*=0.07). The interaction test between PDL1 expression (“PDL1-up” *vs* “PDL1-no up”) and molecular subtypes (basal *vs* non-basal) was significant (*p*=4.8E-04). In multivariate analysis applied to the basal subtype (Table 3A), *PDL1* expression remained the sole prognostic feature for MFS (*p*=1.4E-03, Wald test; HR=0.55 [0.38-0.79]).

**Table 2:** MFS univariate Cox regression analysis in the whole cohort

		N	HR [95CI]	*p*-value
Age (years)	>50 vs ≤50	725	0.85 [0.65-1.12]	0.25
Pathological type	LOB vs DUC	440	1.32 [0.75-2.32]	0.46
	MED vs DUC		0.39 [0.10-1.59]	
	MIX vs DUC		0.65 [0.24-1.79]	
	Other vs DUC		1.14 [0.50-2.61]	
pN	Positive vs Negative	612	1.37 [1.01-1.86]	**4.00E-02**
pT	pT2-4 vs pT1	445	1.77 [1.23-2.56]	**2.23E-03**
SBR grade	2-3 vs 1	857	3.46 [2.08-5.76]	**1.70E-06**
ER status	Positive vs Negative	1080	0.57 [0.47-0.68]	**4.70E-09**
PR status	Positive vs Negative	1080	0.61 [0.5-0.73]	**3.30E-07**
ERBB2 status	Positive vs Negative	1080	1.21 [0.93-1.57]	0.17
*PDL1* expression	“up” vs “no up”	1080	0.94 [0.75-1.17]	0.57
				

N, number of samples with data available; LOB, invasive lobular carcinoma; DUC, invasive ductal carcinoma; MED, medullary carcinoma; MIX, mixt carcinoma (lobular and ductal); pT, pathological tumor size; pN, pathological lymph node involvement; HR, hazard ratio;95CI,95% confidence interval.

**Table 3:** Univariate and multivariate Cox regression analyses for basal tumors

A/ Metastasis-free survival		Univariate analysis			Multivariate analysis		
		N	HR [95CI]	p-value	N	HR [95CI]	*p*-value
Age (years)	>50 vs ≤50	201	1.22 [0.73-2.03]	0.44			
pN	Positive vs Negative	154	1.52 [0.87-2.64]	0.14			
pT	pT2-4 vs pT1	120	1.24 [0.59-2.62]	0.57			
SBR grade	2-3 vs 1	217	0.58 [0.14-2.36]	0.44			
ER status	Positive vs Negative	279	0.46 [0.22-0.94]	**3.00E-02**	279	0.51 [0.25-1.04]	0.06
PR status	Positive vs Negative	279	0.78 [0.48-1.26]	0.31			
ERBB2 status	Positive vs Negative	279	0.43 [0.11-1.76]	0.24			
*PDL1* expression	“up” vs “no up”	279	0.53 [0.36-0.76]	**6.40E-04**	279	0.55 [0.38-0.79]	**1.40E-03**
							

B/ Overall specific survival		Univariate analysis			Multivariate analysis		
		N	HR [95CI]	p-value	N	HR [95CI]	*p*-value
Age (years)	>50 vs ≤50	690	0.84 [0.63-1.12]	0.23			
pN	Positive vs Negative	628	2.15 [1.57-2.93]	**1.50E-06**	483	2.01 [1.43-2.81]	**4.90E-05**
pT	pT2-4 vs pT1	489	1.72 [1.21-2.45]	**2.40E-03**	483	1.46 [1.02-2.08]	**4.00E-02**
SBR grade	2-3 vs 1	630	1.35 [0.43-4.21]	0.61			
ER status	Positive vs Negative	778	0.77 [0.53-1.12]	0.17			
PR status	Positive vs Negative	765	0.68 [0.43-1.07]	0.1			
ERBB2 status	Positive vs Negative	778	1.36 [0.85-2.18]	0.2			
*PDL1* expression	“up” vs “no up”	778	0.52 [0.38-0.71]	**4.20E-05**	483	0.52 [0.35-0.77]	**9.40E-04**

N, number of samples with data available; pT, pathological tumor size; pN, pathological lymph node involvement; HR, hazard ratio; 95CI,95% confidence interval.

### PDL1 expression and overall specific survival

**Figure 3:**
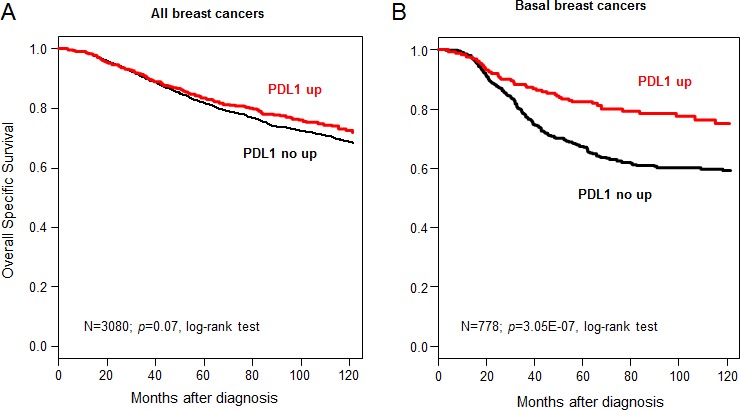
Overall specific survival according to PDL1 mRNA expression in the whole population and in basal breast cancers A/ Kaplan-Meier OSS curves in patients with high and low expression in the whole population. The respective 5-year OSS were 84 and 82%. B/ Similar to (A), but limited to patients with basal breast cancer. The respective 5-year OSS were 82 and 68%.

The results were similar with respect to OSS for the 3,778 patients with available follow-up, including 2,848 who remained alive during a median follow-up of 86 months (median OSS not reached) and 930 who died from disease progression. The 5-year OSS was 82% [95CI = 0.81-0.84]. *PDL1* expression was not associated with OSS in the whole population (*p*=0.07, log-rank test, Figure 3A), but was associated with better OSS for basal tumors, in which the 5-year OSS was 82% (CI95 78-87) in case of *PDL1* upregulation and 68% (CI95 63-72) in the absence of upregulation (p=3.05E-07; log-rank test; Figure 3B). A trend was noted for ERBB2-enriched tumors (*p*=0.07, HR=0.73 [0.52-1.02]), but not for patients from other subtypes (data not shown), and the interaction test between PDL1 expression (“PDL1-up” *vs* “PDL1-no up”) and molecular subtypes (basal *vs* non-basal) was significant (*p*=7.8E-03). Multivariate analysis in basal breast cancers showed that *PDL1* expression remained an independent prognostic feature (*p*=9.4E-04, Wald test; HR=0.52 [0.35-0.77]), as well as pathological tumor size and lymph node status (Table 3B). Given the correlation between *PDL1* expression and the four prognostic immune signatures, we repeated the prognostic analysis for OSS in basal tumors by confronting these five variables. As shown in Table 4, all but one (the immune response module [6]) were associated with OSS in this series. Multivariate analysis including the significant variables showed that *PDL1* expression could independently predict OSS, whereas all gene signatures lost their prognostic value, suggesting dependence on *PDL1* expression.

**Table 4:** OSS Cox regression analyses for basal tumors including four immune response-related gene expression signatures

		Univariate analysis			Multivariate analysis		
		N	HR [95CI]	p-value	N	HR [95CI]	*p*-value
*PDL1* expression	“up” vs “no up”	778	0.52 [0.38-0.71]	**4.23E-05**	778	0.61 [0.43-<0.85]	**3.89E-03**
Immune response module (Teschendorff et al.)	Low-risk vs. high-risk	766	0.86 [0.66-1.13]	0.285			
LCK metagene (Rody et al.)	Low-risk vs. high-risk	778	0.61 [0.46-0.79]	**2.91E-04**	778	0.83 [0.56-1.24]	0.367
Stroma metagene (Bianchini et al.)	Intermediate-risk vs high-risk	778	0.72 [0.53-0.99]	**4.84E-03**	778	0.85 [0.61-1.18]	0.327
	Low-risk vs. high-risk		0.59 [0.42-0.82]		778	0.79 [0.54-1.16]	0.237
Immune kinases metagene (Sabatier et al.)	Low-risk vs. high-risk	778	0.61 [0.45-0.84]	**2.05E-03**	778	0.92 [0.6-1.42]	0.702

N, number of samples with data available; HR, hazard ratio; 95CI,95% confidence interval.

### PDL1 expression and pathological response to chemotherapy

Pathological response after neoadjuvant anthracycline-based chemotherapy was available for 265 out of 5,454 patients. Seventy-six (29%) of these patients displayed pathological complete response (pCR), and 189 did not. In this pooled series, *PDL1* expression was associated with pCR in the whole population (50% pCR in case of upregulation *versus* 21% in the other cases; *p*=6.6E-06, OR=3.8 [2.05-7.09]), whereas none of the tested clinicopathological features did (Table 5, data related to grade could not be interpreted because only 4 (2%) grade 1 tumors were included, all presenting a residual disease after chemotherapy). We confirmed the predictive value of *PDL1* expression in basal (*p*=3.8E-04, OR=4.3 [1.80-10.43]) and ERBB2-enriched cases (*p*=0.04, OR=6.5 [1.06-50.85]), but not in the other subtypes (luminal A, *p*=0.15; luminal B, *p*=1; normal-like, *p*=0.30).

**Table 5:** Univariate analysis for pathological response to neoadjuvant chemotherapy in the whole cohort and per molecular subtype

		N	RD	pCR	*p*-value*	OR [95CI]
All variables and PDL1 expression in the whole cohort	PDL1 expression				6.60E-06	3.8 [2.05-7.09]
	‘no up’	193	153 (81%)	40 (53%)		
	‘up’	72	36 (19%)	36 (47%)		
	Age (years)				0.5	0.82 [0.46-1.45]
	≤50	151	105 (56%)	46 (61%)		
	>50	114	84 (44%)	30 (39%)		
	Pathological type				0.27	
	DUC	115	67 (94%)	48 (86%)		
	LOB	6	2 (3%)	4 (7%)		
	Other	6	2 (3%)	4 (7%)		
	ER status				0.056	3.1 [0.85-11.51]
	Negative	252	183 (97%)	69 (91%)		
	Positive	13	6 (3%)	7 (9%)		
	PR status				0.87	1.1 [0.53-2.12]
	Negative	208	149 (79%)	59 (78%)		
	Positive	57	40 (21%)	17 (22%)		
	ERBB2 status				0.32	1.4 [0.68-2.68]
	Negative	209	152 (80%)	57 (75%)		
	Positive	56	37 (20%)	19 (25%)		
PDL1 expression in each molecular subtype	Basal				3,76E-04	4.3 [1.8-10.43]
	‘no up’	92	76 (77%)	16 (43%)		
	‘up’	44	23 (23%)	21 (57%)		
	ERBB2-enriched				3,78E-02	6.5 [1.06-50.<85]
	‘no up’	31	24 (89%)	7 (54%)		
	‘up’	9	3 (11%)	6 (46%)		
	Luminal A				0.15	2.8 [0.54-14.26]
	‘no up’	40	31 (84%)	9 (64%)		
	‘up’	11	6 (16%)	5 (36%)		
	Luminal B				1.00	[0.02-156.46]
	‘no up’	11	7 (88%)	4 (80%)		
	‘up’	2	1 (12%)	1 (20%)		
	Normal-like				0.30	3.5 [0.34-38.7]
	‘no up’	19	15 (83%)	4 (57%)		
	‘up’	6	3 (17%)	3 (43%)		
	

N, number of samples with data available; RD, residual disease; pCR, pathological complete response; DUC, invasive ductal carcinoma; LOB, invasive lobular carcinoma; OR, odd ratio; 95CI, 95% confidence interval.

* Fisher's exact test

### PDL1 expression and associated biological processes

Supervised analysis identified 359 genes differentially expressed in the Guedj's dataset between the tumors with *versus* without *PDL1* upregulation, including 287 genes upregulated and 72 genes downregulated in the “PDL1-up” samples (Supplementary Table 5, Supplementary Figure 3). The robustness of this gene list was confirmed in two large independent sets of respectively 448 and 533 tumors by using a metagene approach and ROC curves with “PDL1-up” genes (Supplementary Figure 3) with respective accuracy rates of 90% (p=2.6E-49, Fisher's exact test) and 80% (p=1.4E-50, Fisher's exact test). Ontology analysis of these 359 genes (Supplementary Table 6) revealed that “PDL1-up” tumors overexpressed genes involved in the regulation of the local immune response. More specifically, we found that numerous upregulated genes were related to the T-cell receptor (*TCR alpha, beta, delta*, *CD247*, *CD2*, *KLRK1*, *CD8A*, *PTPRC*, *CD3D*, *CD3E*,…), attesting of a high infiltration with T-cells in the “PDL1-up” group. In addition, two of the most overexpressed genes in the “PDL1-up” group were *IDO1* and *CTLA4,* known as major actors induced to attenuate an active T-cell immune response. However, most of the other genes associated with PDL1 upregulation were genes directly involved in T-cells activation (*ZAP70*, *ITK*, *LCK*, *JAK3*,…), differentiation factors (*EOMES*, *STAT1*, *STAT4*, *CD27*…), cytotoxic effector molecules (*GZMA*, *GZMB*, *GZMK*, *PRF1*, *GNLY*, *C1QA*…), inflammation/anti-tumor cytokines (*IL2RG*, *IL2RB*, *IL21R*, *IL27R*, *IL15*, *IL18BP*, *LTB*, some interferon-induced proteins…), and chemokines related to T-cells activation and homing (*CCL2*, *CCL4*, *CCL5*, *CCL8*, *CCL18*, *CXCL1*, *CXCL9-11*, *CCR5*,…). This signature was highly suggestive of a major anti-tumor immune response occurring at the tumor site in the “PDL1-up” group. Many genes overexpressed in the “PDL1-no up” group were involved in response to hormone stimulus and mammary gland development and luminal differentiation such as *ESR1*, *TTF1*, *GATA3* and *ERBB4*.

## DISCUSSION

Blockade of the PD1-PDL1 pathway is a new promising therapeutic approach in oncology. Our objective was to document the expression of *PDL1* in a large series of breast cancer cell lines and clinical samples and to search for correlations with tumor features. We showed that *PDL1* expression was associated with more aggressive subtypes (basal and ERBB2-enriched). In basal tumors, higher *PDL1* expression was associated with better MFS and OSS and better response to chemotherapy. To our knowledge, this is the largest series reported with more than 5,400 cases analyzed.

During the last decade, PDL1 expression in cancer has been mainly studied at the protein level using IHC, but divergent results have been reported, notably regarding its prognostic value [41]. These divergences have often been related to the absence of standardization of PDL1 IHC, notably in terms of specificity and reproducibility of available antibodies [42, 43], definition of optimal positivity cut-off and interpretative subjectivity. Alternative analytic methods have been developed, such as mRNA analysis using *in situ* hybridization (ISH) [36]. In breast cancer, *PDL1* mRNA expression measured using an antibody-independent ISH assay was associated with a long recurrence-free survival [36], whereas protein expression measured using IHC with a commercial rabbit polyclonal antibody was associated with a poor survival [39]. In fact, this rabbit antibody, as well as other commercial clones, failed validation using Western blot and IHC [41, 43]. Similarly, the use of a validated monoclonal antibody (clone 5H1) provided a favorable prognostic value in lung [41, 44] and colorectal [31, 45] carcinomas, opposite to what had been previously reported with non-validated antibodies. Our analysis at the mRNA level and based on DNA microarrays allowed us to avoid the limitations of IHC and to work on a very large series of samples, providing for the first time the opportunities to address different clinical issues (survival, response to chemotherapy) and to work on each molecular subtype separately. One limitation of DNA microarray-based measurement is that it quantifies expression level of both tumor cells and non-tumor cells, including immune infiltrating cells. However, our results are consistent with those reported by others using ISH [36], who showed that the hybridization signal in breast cancer samples was predominantly located within the tumor cells.

We found *PDL1* upregulation in 20% of 5,454 breast cancers and 38% of 1,205 basal tumors. To date, five teams have described PDL1 expression in breast cancer [33-39]. The first study reported expression in 50% of 44 samples analyzed using IHC and a little stringent positivity cut-off (expression by at least one cell) [34]. Using different antibodies and scoring systems, Muenst *et al* reported PDL1 expression in ~23% of 650 samples [39], and Mittendorf *et al* in 19% of 105 triple-negative (TN) samples [38]. Using ISH, Schalper *et al* reported *PDL1* mRNA expression - defined as signal detection as compared to a negative control bacterial gene - in ~55% of 636 samples [36]. The relative lower frequency of upregulation that we found (20%) may account for the different scoring system that we used (upregulation defined by an arbitrary cut-off: T/NB ratio ≥2) and the different analytic levels, protein *versus* mRNA, that show a positive but non-linear relationship [36]. In addition to mRNA upregulation, we searched for other molecular alterations of PDL1 in breast cancer and showed for the first time that copy number alterations are rare (<5%), even if this rate was higher in basal tumors and correlated with a higher rate of mRNA overexpression. Similarly, analysis of the TCGA data set [46] shows that mutations are very rare with only one tumor mutated out of 464 tested (0.2%).

Analysis of correlations between *PDL1* mRNA expression and tumor features, showed that, in agreement with previous publications on smaller series [34, 35, 39], *PDL1* upregulation was associated with poor-prognosis features: large tumor size, high grade, negative ER status, negative PR status, positive *ERBB2* status, and high proliferation rate. For the first time, we showed that *PDL1* was differentially expressed across the major breast cancer molecular subtypes, with more frequent upregulation in basal and ERBB2-enriched subtypes than in luminal A, luminal B and normal-like subtypes. Recently, greater expression was reported in TN *versus* non-TN breast cancers [37, 38]. The results on cell lines were similar with *PDL1* overexpression in basal and mesenchymal lines as compared to luminal lines, as previously reported [37]. The correlation between *PDL1* upregulation and elevated tumor cell proliferation (Ki67) and more proliferative molecular subtypes might be explained by the higher mutation rate of hyperproliferative tumor cells, potentially responsible for higher immunogenicity due to the rapid appearance of neoantigens. Of note, all these correlations persisted when *PDL1* expression was analyzed as continuous value (data not shown).

A total of 1,080 cases were informative for MFS and 3,778 for OSS. *PDL1* upregulation was not associated with survival in the whole series, nor in the other subtypes than basal, but was associated with better MFS and OSS in basal breast cancers. In both cases, multivariate analyses showed that *PDL1* had independent prognostic value. Of note, we found similar prognostic results (data not shown) when *PDL1* expression was analyzed as continuous value and when the molecular subtypes were defined using mRNA expression of ER, PR and ERBB2. *PDL1* upregulation was a favorable independent prognostic variable for MFS (*p*=5.1E-05, Wald test) and OSS (*p*=0.03, Wald test) in the TN subtype, whereas no prognostic value was found in univariate analysis in the ER+ and/or PR+/ERBB2- and ERBB2+ subtypes. To date, only two breast cancer studies have reported significant and independent prognostic value of PDL1 expression in smaller series than ours [36, 39]. Analyses were done in the whole series of samples, and not per molecular subtype, but with contradictory results: mRNA expression (ISH assay; N=398) was associated with longer recurrence-free survival [36] in agreement with our observation, whereas protein expression (IHC with a commercial antibody; N=650) was associated with worse overall survival [39]. Possible explanations for these discordances may be related to the IHC limitations described above and the non-perfect correlation between mRNA and protein expression [36]. In fact, recent studies using validated IHC assays in different cancers showed that PDL1 expression had a favorable prognostic impact [31, 41, 47], by contrast to what had been previously suggested with non-validated antibodies. We previously found counterintuitive similar favorable prognostic value with IDO expression in breast cancer using both mRNA and IHC analyses [48].

Such favorable prognostic value seems paradoxical given the known immunosuppressive role of PDL1 whose up-regulation in clinical samples is usually associated with immune features suggestive of anti-tumor escape mechanism. The biological explanation for our observation might be that *PDL1* expression is rather a marker of engaged CD8+ TILs, known to provide favorable prognostic features [2, 3], and represents a negative feedback mechanism (like IDO overexpression) that followed the CD8+ infiltration [32, 49]. Indeed, we observed a robust immune signature in the “PDL1-up” group. The genes associated with this immune signature were characteristic of a strong cytotoxic response, involving CD8+ T-cells, but also other actors of anti-tumor immunity (γδ-T-cells, NKG2D+ cells, dendritic-cells, B-cells …). This activation profile was consistent with the correlation between *PDL1* mRNA expression and the presence of elevated TILs reported by other groups on smaller series [33, 36]. While associated with other immunosuppressive molecules of the “PDL1-up” group, such as IDO and CTLA4, this infiltrate was however highly suggestive of an activated profile of differentiated T-cells (EOMES, CD27…). Those cells were clearly T_H_1-biased (IL12 and IFN-induced pathways), endowed with cytotoxic effector functions (granzymes, perforine, granulysine). In addition, this pro-cytotoxic profile was coherent with our description of the positive correlation between *PDL1* transcript and known immune expression signatures [4-7], as well as the probability of activation of IFNα, IFNγ, STAT3 and TNFα pathways [40]. In addition, none of the characteristic markers related to T-cell exhaustion (TIM3, LAG3, BTLA) were up-regulated in the “PDL1-up” group. Altogether, these observations suggested that the biological link between *PDL1* upregulation and activated T-lymphocyte infiltrate might be related to IFNγ or other inflammatory cytokines, secreted by anti-tumor T_H_1-cells or macrophages, which can positively regulate PDL1 expression, notably in basal tumor cells [37] in response to immune-mediated attack [19], in order to decrease the cytotoxic local immune response. From a therapeutic point of view, the blockade of PDL1 would allow to reactivate inhibited T-cells to increase the anti-tumor immune response, explaining the benefit observed in responder patients. Interestingly our results of multivariate analysis might suggest that *PDL1* expression reflects more precisely the degree of TILs functionally engaged than immune gene expression signatures.

Finally, *PDL1* upregulation was also associated with better response to pre-operative chemotherapy in the 265 informative samples, with a 50% pCR rate *versus* 21% in case of no upregulation. Per molecular subtype analysis showed that this predictive value was in fact limited to basal and ERBB2-enriched tumors. Analysis of *PDL1* expression as continuous value gave the same results (data not shown). Like the prognostic correlation, this rather counterintuitive correlation has likely the same biological explanation and is likely related to the known favorable predictive value of TILs in breast cancer [50-53]. To our knowledge, this is the first demonstration of such a correlation in breast cancer.

In conclusion, we showed that *PDL1* mRNA expression is associated with basal subtype where it represents an independent favorable prognostic feature and a predictive feature for better response to chemotherapy. The main strength of our study lies in the number of samples analyzed (more than 5,400), allowing both overall and per subtype prognostic and predictive analyses. Limitations include its retrospective nature, the absence of information with respect to survival and response to chemotherapy for more samples, and the use of DNA microarrays that quantify expression level of both epithelial and stromal cells. However, our study, together with the known link between PDL1 expression and tumor response to PDL1-inhibitors, suggests that the therapeutic targeting of PDL1 in basal breast cancers could enhance the local immune response, thus providing an antitumor effect and decreasing the metastatic risk and improving the therapeutic response when associated with immunogenic anticancer chemotherapy such as doxorubicin [54, 55]. Functional and clinical validation of this hypothesis is urgently warranted.

## MATERIALS and Methods

### Breast cancer samples

Breast cancer cell lines and clinical samples were profiled using DNA microarrays. Our own data set of breast cancer cell lines included 45 cell lines: BT-20, BT-474, BT-483, CAMA-1, HBL100, HCC38, HCC202, HCC1395, HCC1500, HCC1569, HCC1806, HCC1937, HCC1954, HME-1, carcinosarcoma-derived Hs578T, MCF-7, MCF-10A, MDA-MB-134, MDA-MB-157, MDA-MB-175, MDA-MB-231, MDA-MB-361, MDA-MB-415, MDA-MB-436, MDA-MB-453, SK-BR-3, SK-BR-7, T47D, UACC-812, ZR-75–1, ZR-75–30 (http://www.atcc.org/), HMEC-derived 184A1 and 184B5 (ATCC, http://www.atcc.org/), BrCa-MZ-01, SUM44, SUM-52, SUM102, SUM-149, SUM159, SUM-185, SUM-190, SUM206, SUM-225, SUM229 http://www.cancer.med.umich.edu/breast_cell/production), and S68 (a kind gift from V. Catros, Cell Biology Department, CHU Rennes, France). All cell lines are derived from carcinomas except MCF-10A, which is derived from a fibrocystic disease, and HME-1 and 184B5, which represent normal mammary tissue.

Our own data set of clinical samples included 286 cases representing pre-treatment invasive carcinomas from patients with non-metastatic and non-inflammatory disease at diagnosis. The study was approved by our institutional review board (the Institut Paoli Calmettes (IPC) “Comité d'Orientation Stratégique”) and each patient had given a written informed consent for research use. We pooled it with 17 available data sets comprising at least one probe set representing *PDL1*. These sets were collected from the National Center for Biotechnology Information (NCBI)/Genbank GEO and ArrayExpress databases, and authors' website (Supplementary Table 2). The final pooled data set included 5,454 non-redundant non-metastatic, non-inflammatory, primary, invasive breast cancers with *PDL1* mRNA expression and clinicopathological data available.

Five out of the 18 data sets included also array-comparative genomic hybridization (aCGH) data (Supplementary Table 2), corresponding to 3,140 breast cancer samples with available whole-genome DNA copy number alterations.

### Gene expression data analysis

Our own gene expression data set (cell lines, clinical normal and cancer samples) had been generated using Affymetrix U133 Plus 2.0 human microarrays (Affymetrix®, Santa Clara, CA, USA) as previously described [56]. All data were MIAME compliant and deposited in the Array-Express and GEO databases (E-MTAB-1693 and GSE31448).

The breast cancer cell lines and clinical samples were analyzed separately. Cell lines were analyzed as previously described [57]. *PDL1* expression was measured by analyzing different probe sets whose identity and specificity were verified using the NCBI program BLASTN 2.2.29+ (Supplementary Table 1). The molecular subtypes (luminal, basal, and mesenchymal) were defined as previously reported [58]. Seventeen cell lines were classified as luminal, 11 as basal, and 8 as mesenchymal, whereas 9 could not be classified in any subtype.

Data analysis of clinical samples required pre-analytic processing. The first step was to normalize each data set separately: we used quantile normalization for the available processed data from non-Affymetrix-based sets (Agilent, SweGene and Illumina), and Robust Multichip Average (RMA) [59] with the non-parametric quantile algorithm for the raw data from the Affymetrix-based data sets. Normalization was done in R using Bioconductor and associated packages. Then, hybridization probes were mapped across the different technological platforms represented. We used SOURCE (http://smd.stanford.edu/cgi-bin/source/sourceSearch) and EntrezGene (Homo sapiens gene information db, release from 09/12/2008, ftp://ftp.ncbi.nlm.nih.gov/gene/) to retrieve and update the non-Affymetrix gene chips annotations, and NetAffx Annotation files (www.affymetrix.com; release from 01/12/2008) to update the Affymetrix gene chips annotations. The probes were then mapped based on their EntrezGeneID. When multiple probes mapped to the same GeneID, we retained the one with the highest variance in a particular dataset.

To avoid biases related to IHC analyses across different institutions and thanks to the bimodal distribution of respective mRNA expression levels, ER, progesterone receptor (PR), ERBB2 and Ki67 expression (negative/positive) was defined at the transcriptional level using gene expression data of *ESR1, PGR, ERBB2* and *MKI67* respectively, as previously described [60]. We applied different multigene classifiers in each data set separately. The intrinsic molecular subtypes of tumors were defined using the PAM50 classifier [61] as previously described [62]. Out of the 5,454 analyzed breast cancers, 1,515 were identified as luminal A (28%), 1,243 as luminal B (23%), 1,205 as basal (22%), 841 as ERBB2-enriched (15%), and 650 as normal-like (12%). Clinicopathological characteristics of subtypes are summarized in Supplementary Table 3. Because of the involvement of PDL1 in immunity, we also analyzed prognostic gene expression signatures (GES) linked to immune response [4-7]. Since these GES were validated for ER negative, triple negative or basal-like tumors, we compared their prognostic value to that of PDL1 only for basal-like cases. We also assessed the correlation between *PDL1* expression and previously published GES of biological pathway activity [40].

Before analysis of *PDL1* mRNA expression, gene expression data were standardized within each data set using the luminal A population as reference. This allowed to exclude biases due to laboratory-specific variations and to population heterogeneity and to make data comparable across all sets. A principal component analysis (PCA) applied to the 5,454 tumors and the genes of PAM50 signature prior and after the standardization allowed to verify the accuracy of the normalization in removing the set-specific variation in gene expression (Supplementary Figure 4): the samples, initially grouped according to their data set of origin (before standardization), were grouped according to their molecular subtype after standardization, suggesting that the normalization maintained the biological information included in the expression data. *PDL1* expression in tumors (T) was measured as discrete value after comparison with mean expression in normal breast samples (NB): upregulation, thereafter designated “up” was defined by a T/NB ratio ≥2 and no upregulation (“no up”) by a T/NB ratio <2.

To explore the biological pathways linked to *PDL1* expression in breast cancer, we applied a supervised analysis to three large data sets including more than 400 samples: the Guedj' s data set [63] as learning set, including 100 tumors with and 352 without *PDL1* upregulation, and the Ivshina' s data set [64] and the TCGA data set [46] as two independent validation sets, including respectively 112 tumors with and 336 without *PDL1* upregulation, and 123 tumors with and 410 without *PDL1* upregulation. In the learning set, we compared the expression profiles of 16,578 genes between tumors with *versus* without *PDL1* upregulation using a moderated t-test with the following significance thresholds: p<5%, q<5% and fold change (FC) superior to |2x|. Ontology analysis of the resulting gene list was based on GO biological processes of the Database for Annotation, Visualization and Integrated Discovery (DAVID; david.abcc.ncifcrf.gov/). We tested the robustness of the resulting gene list on each validation set separately by computing for each tumor sample a “metagene PDL1 up” as the mean expression of all genes identified as upregulated in the “PDL1 up” group compared to the “PDL1 no up” group. The metagene threshold that defined a sample as “PDL1 up” or “PDL1 no up” was determined on a receiver operating characteristic (ROC) curve applied to the learning set. The metagene was applied to the validation sets to define the predicted PDL1 expression status of each sample. Correlation of the predicted status with the observed status was assessed using ROC curve of the “metagene PDL1 up” using Fisher's exact test.

### Array-CGH (aCGH) data analysis

Genomic imbalances, associated with available mRNA expression data, were available for 3,140 breast cancer samples extracted from 5 sets including ours [46, 65-68] (Supplementary Table 2). Data from our set were generated using 244K CGH Microarrays (Hu-244A, Agilent Technologies Inc. Santa Clara, CA, USA) as previously described [65, 69]. The *PDL1* locus at 9p24 was analyzed, and copy number changes were characterized as reported previously [65]. Five probes (A_16_P02052215, A_16_P02052226, A_16_P18538134, A_14_P120124 and A_16_P02052257) matched the *PDL1* gene on our Agilent chips. All our aCGH data can be found in the Array Express database (E-MTAB-1861) and the GEO database (GSE23720). DNA copy number alterations in tumors were defined as a 1.5 FC for gains and losses, and a 2 FC for amplifications and deletions as compared to normal DNA. Non-segmented aCGH data [66, 70] were processed as previously described [70] using circular binary segmentation (CBS).

### Statistical analysis

Correlations between tumor groups and clinicopathological features were analyzed using the t-test or the Fisher's exact test (variables with 2 groups) when appropriate or one-way analysis of variance (ANOVA; variables with more than 2 groups). Metastasis-free survival (MFS) was calculated from the date of diagnosis until the date of distant relapse. Overall specific survival (OSS) was calculated from the date of diagnosis to the date of death from breast cancer. Follow-up was measured from the date of diagnosis to the date of last news for event-free patients. Survivals were calculated using the Kaplan-Meier method and curves were compared with the log-rank test. Univariate and multivariate survival analyses were done using Cox regression analysis (Wald test). Variables tested in univariate analyses included patients' age at time of diagnosis (≤50 years *vs* >50), pathological tumor size (pT: pT1 *vs* pT2-4), pathological axillary lymph node status (pN: negative *vs* positive), pathological grade (1 *vs* 2-3), histological type, and *PDL1* expression (“up” *vs* “no up”). Variables with a *p*-value <0.05 in univariate analysis were tested in multivariate analysis. Differences in the prognostic effect of *PDL1* expression by molecular subtype were assessed using a Cox model with an interaction term between expression and subtype. We also analyzed the pathological response after neoadjuvant chemotherapy which was available for 265 clinical samples: pathological complete response (pCR) was defined as absence of invasive cancer in both breast and axillary lymph nodes. All statistical tests were two-sided at the 5% level of significance. Statistical analysis was done using the survival package (version 2.30) in the R software (version 2.9.1; http://www.cran.r-project.org/). We followed the reporting REcommendations for tumor MARKer prognostic studies (REMARK criteria) [71].

## SUPPLEMENTARY TABLES














