# Supplementary material for: Prognostic and predictive value of PDL1 expression in breast cancer
Source: Oncotarget. 2014 Dec 31;6(7):5449–64. doi: 10.18632/oncotarget.3216 (PMC4467160; doi:10.18632/oncotarget.3216)
Supplement: Supplementary file 1 [file oncotarget-06-5449-s001.pdf]

Prognostic and predictive value of PDL1 expression in breast cance

Formatted: Left: 1", Right: 1", Top: 1", Bottom: 1", Width: 8.5", Height: 11", Header distance from edge: 0.5", Footer distance from edge: 0.5"

Supplementary Material

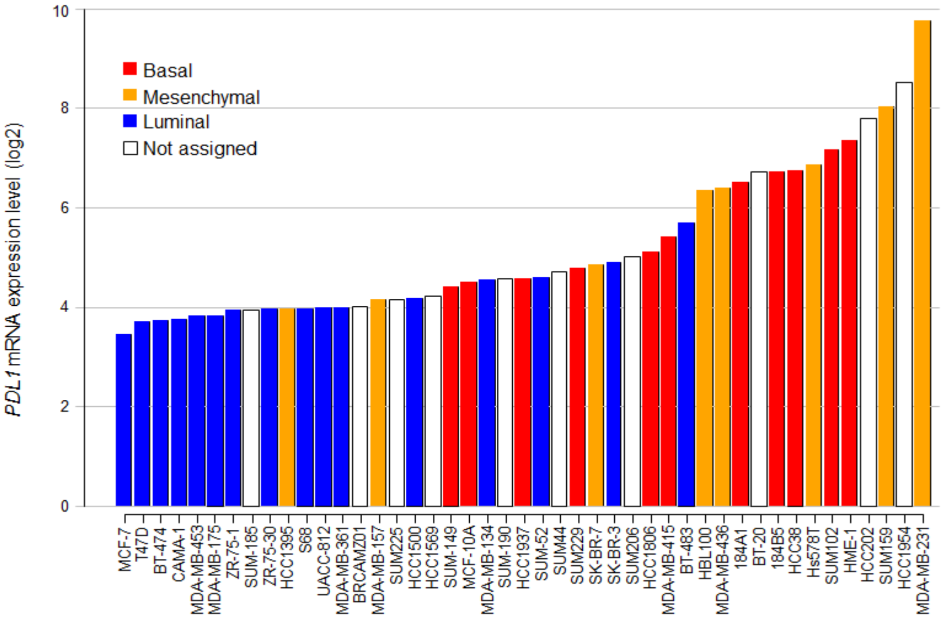

Supplementary Figure 1: *PDL1* expression in 45 profiled mammary cell lines

mRNA expression is shown in log2 scale. The molecular subtype of each cell line is color-coded as indicated.

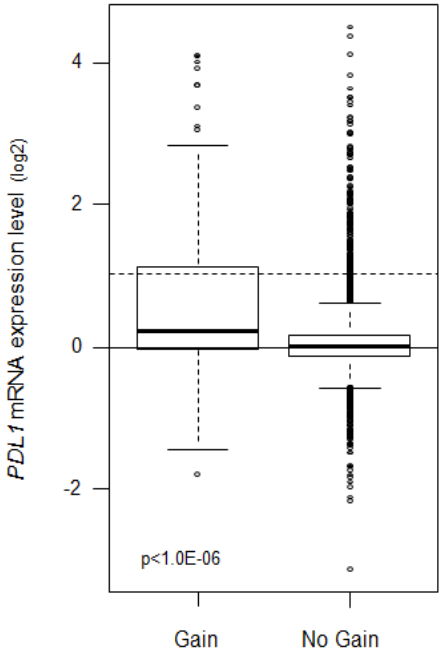

Supplementary Figure 2: Correlation between *PDL1* DNA copy number and mRNA expression in 3,140 clinical breast cancer samples

Box plot of *PDL1* expression in shown according to *PDL1* genomic status, with (left) and without (right) gain defined as a DNA copy number ratio tumor/NB ≥1.5 (horizontal dashed line). Difference in *PDL1* expression levels between both groups was tested for significance using Student t-test. For each box plot, median and range are indicated.

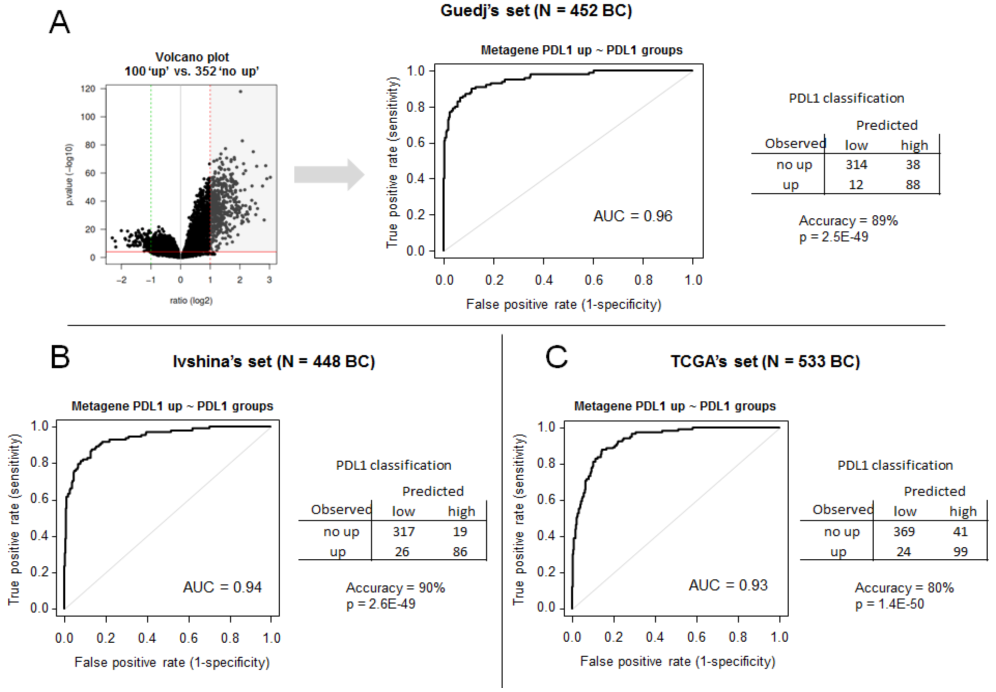

Supplementary Figure 3: Identification and independent validation of the PDL1-up gene expression signature

A/ Identification of the signature in Guedj's set. *Left*, Volcano-plot showing the 359 genes differentially expressed between breast cancers with *versus* without *PDL1* upregulation. *Middle*, ROC curve of the "metagene PDL1 up". The high area under curve (AUC=0.96) reflects the strong and expected positive correlation between the predicted and observed *PDL1* expression status. *Right*, cross-table between the two classifications (Fisher's exact test) **B/** Ivshina's validation set. *Left*, ROC curve of the "metagene PDL1 up". The high area under curve (AUC=0.94) reflects the strong positive correlation between the predicted and observed *PDL1* expression status. *Right*, cross-table between the two classifications (Fisher's exact test). **C/** Similar to B, but in the TCGA validation set.

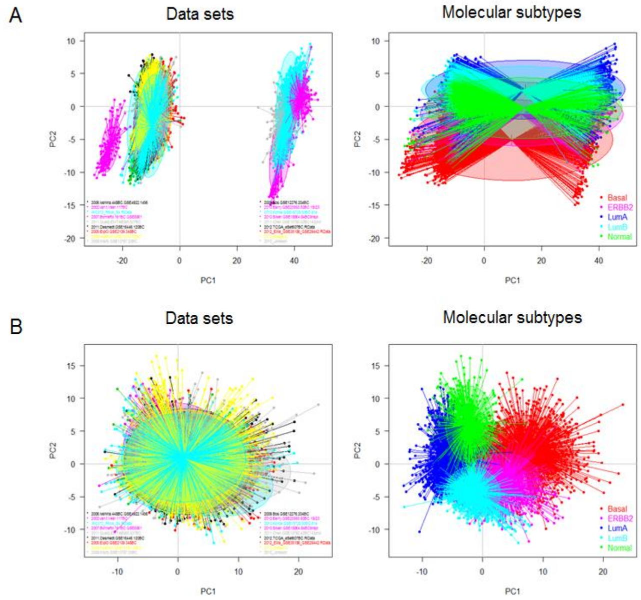

Supplementary Figure 4: Principal Component Analysis (PCA) of breast cancers and PAM50 genes before and after standardization

PCA was applied to the 5,454 breast cancer samples and the 36/50 PAM50 genes common to all data sets. **A/** Before standardization, breast cancer samples in the 2D scatter plot representation are grouped according to their origin data set (*left*: each color represents a set) and not according to the molecular subtypes (*right*: dark blue for luminal A, light blue for luminal B, red for basal, pink for ERBB2-overexpressing, and green for normal-like). **B/** After standardization, samples are correctly clustered according to their molecular subtypes (*right*), rather than their origin data set is observed (*left*), clearly suggesting that the standardization has removed technical differences in gene expression while maintaining the information relevant to biological differences.

Supplementary Table 1: List of *PDL1* probe sets analyzed

Supplementary Table 2: List of breast cancer data sets included

Supplementary Table 3: Patients and tumor characteristics

Supplementary Table 4: *PDL1* expression and immune response-related gene expression signatures

Supplementary Table 5: List of 359 genes differentially expressed between breast cancers with *versus* without *PDL1* upregulation

Supplementary Table 6: GO biological processes associated with the 359 genes differentially expressed between breast cancers with *versus* without *PDL1* upregulation
